# Finasteride Inhibits Human Prostate Cancer Cell Invasion through MMP2 and MMP9 Downregulation

**DOI:** 10.1371/journal.pone.0084757

**Published:** 2013-12-30

**Authors:** Andrei Moroz, Flávia K. Delella, Rodrigo Almeida, Lívia Maria Lacorte, Wágner José Fávaro, Elenice Deffune, Sérgio L. Felisbino

**Affiliations:** 1 Univ Estadual Paulista – UNESP, Institute of Biosciences, Department of Morphology, Extracellular Matrix Lab, Botucatu, São Paulo, Brazil; 2 Univ Estadual Paulista - UNESP, Botucatu Medical School, Blood Transfusion Center, Cell Engineering Lab, Botucatu, São Paulo, Brazil; 3 Univ Campinas - UNICAMP, Institute of Biology, Department of Structural and Functional Biology, Campinas, São Paulo, Brazil; 4 Univ Estadual Paulista - UNESP, Botucatu Medical School, Department of Urology, Botucatu, São Paulo, Brazil; Thomas Jefferson University, United States of America

## Abstract

**Introduction:**

The use of the 5-alpha reductase inhibitors (5-ARIs) finasteride and dutasteride for prostate cancer prevention is still under debate. The FDA recently concluded that the increased prevalence of high-grade tumors among 5-ARI-treated patients must not be neglected, and they decided to disallow the use of 5-ARIs for prostate cancer prevention. This study was conducted to verify the effects of finasteride on prostate cell migration and invasion and the related enzymes/proteins in normal human and tumoral prostatic cell lines.

**Materials and Methods:**

RWPE-1, LNCaP, PC3 and DU145 cells were cultivated to 60% confluence and exposed for different periods to either 10 µM or 50 µM finasteride that was diluted in culture medium. The conditioned media were collected and concentrated, and MMP2 and MMP9 activities and TIMP-1 and TIMP-2 protein expression were determined. Cell viability, migration and invasion were analyzed, and the remaining cell extracts were submitted to androgen receptor (AR) detection by western blotting techniques. Experiments were carried out in triplicate.

**Results:**

Cell viability was not significantly affected by finasteride exposure. Finasteride significantly downregulated MMP2 and MMP9 activities in RWPE-1 and PC3 cells and MMP2 in DU145 cells. TIMP-2 expression in RWPE-1 cells was upregulated after exposure. The cell invasion of all four tested cell lines was inhibited by exposure to 50 µM of finasteride, and migration inhibition only occurred for RWPE-1 and LNCaP cells. AR was expressed by LNCaP, RWPE-1 and PC3 cells.

**Conclusions:**

Although the debate on the higher incidence of high-grade prostate cancer among 5-ARI-treated patients remains, our findings indicate that finasteride may attenuate tumor aggressiveness and invasion, which could vary depending on the androgen responsiveness of a patient’s prostate cells.

## Introduction

Prostate cancer is the most common malignancy in men and accounts for $8 billion and an average cost of $81658 per patient, from diagnosis to death, in the USA [1]. A number of agents are currently being investigated for the prevention of prostate cancer [2]. Finasteride, a type 2 5-alpha reductase inhibitor (5-ARI) that blocks the conversion of testosterone (T) into dihydrotestosterone (DHT) [3], is a well-known drug that is used for the treatment of benign prostatic hyperplasia [4] and has been suggested to act as a chemopreventive agent for prostate cancer. The Prostate Cancer Prevention Trial (PCPT) demonstrated a 24.8% reduction in overall and low-grade prostate cancer risk with the administration of finasteride. However, high-grade cancers were noted in 6.4% of finasteride-treated patients, compared to 5.1% of men who received a placebo [5], [6]. This finding led to an important question: did finasteride induce high-grade cancer or increase its detection? This question was followed by an intense debate about factual or artifactual overestimation of high-grade cases in the finasteride-treated patients [3], [7], which divided urologists and prostate researchers. More recently, the REDUCE trial reported similar outcomes after 5-ARI dutasteride treatment. Recognizing the importance of this issue, the Food and Drug Administration (FDA) has recently reanalyzed the data from the PCPT and REDUCE trials and concluded that finasteride and dutasteride treatments might increase the risk of a more serious form of prostate cancer. Therefore, they decided to disallow the use of these agents for prostate cancer prevention [8]. In addition, a recently published experimental study revealed similar outcomes to the PCPT and REDUCE trials [9]. The authors demonstrated that the incidence of poorly differentiated carcinoma was increased in C57BL/6 TRAMP × FVB mice fed with a finasteride supplemented diet, and considered this as an adverse effect of finasteride treatment, rather than an artifactual effect [9].

High-grade prostate cancer cases, such as those observed in the 5-ARI-treated patients, are commonly associated with an increased expression of matrix metalloproteinases (MMPs), a family of zinc and calcium dependent endopeptidases that are responsible for extracellular matrix (ECM) remodeling, which contributes to invasive and metastatic phenotypes of prostate cancer cells [10]–[13], and decreased expression of tissue inhibitor of matrix metalloproteinases (TIMPs) [13], a class of naturally occurring inhibitors of MMPs that tightly regulate their activity and are expressed in a variety of cell types [11].

Because ECM degradation is known to be a major step during cancer progression [10], [12], [13], our group has been investigating the effects of finasteride upon MMP and TIMP modulation in an attempt to explain why finasteride-treated patients had higher-grade prostate cancers. We previously demonstrated that finasteride treatment increased the expression of MMP9 and decreased the expression of MMP2 in the rat ventral prostate [14], [15] and that it downregulated the mRNA levels of TIMP-1 and TIMP-2 in the rat ventral prostate [15]. Moreover, we have recently demonstrated that finasteride also reduces the MMP2 gelatinolytic activity in a variety of human prostate cell lines [16]. We conducted the present study to ascertain whether finasteride treatment interferes with the migration and invasive potential of the normal human prostate cell line RWPE-1 and the tumoral epithelial cell lines LNCaP, PC3 and DU145, which have different androgen receptor (AR) profiles.

## Materials and Methods

### Cell Cultures

One vial each of the RWPE-1 (non-tumoral, wild type AR^+^), LNCaP (tumoral, mutated AR^+^) and PC3 (tumoral, AR^−^) cell lines were acquired from the American Type Culture Collection (ATCC™). DU145 (tumoral, AR^−^) cells were kindly donated by Dr. Heloisa Sobreiro Selistre de Araújo from the Department of Physiology – UFSCar, Brazil (originally acquired from the ATCC™). Although not isogenic, these cell lines were selected to be representative of 3 distinct in vivo finasteride exposure situations: a patient with a normal prostate (represented in vitro by the RWPE-1 cell line), a patient with androgen-sensitive prostate cancer (represented in vitro by the LNCaP cell line) and a patient with castration-resistant prostate cancer (represented in vitro by the PC3 and DU145 cell lines). The experiments were performed two months after the acquisition of the cell lines. The RWPE-1 cells were cultivated using Keratinocyte Serum Free Medium (Invitrogen™) supplemented with 0.05 mg/ml bovine pituitary extract, 5 ng/ml recombinant human epidermal growth factor (EGF) and 1% antibiotic/antimycotic solution (Invitrogen™). The LNCaP, PC3 and DU145 cells were cultivated using RPMI medium (Invitrogen™) supplemented with 10% fetal bovine serum (SFB) (Invitrogen™) and 1% antibiotic/antimycotic solution (Invitrogen™). All cell culture procedures were performed under strict sterile conditions and kept inside a 5% CO_2_ incubator (Thermo Scientific™). The initial vial of each cell line was expanded up to 10 vials and individually cryopreserved in the respective cell culture medium supplemented with 20% FBS and 10% dimethylsulfoxide (DMSO) (Sigma-Aldrich™) to constitute the cell bank.

### Finasteride Exposure and Cell Viability

All cell lines were then thawed and individually seeded at 2×10^4^ cells/cm^2^ in 6-well culture plates (TPP™). Experiments were carried out in triplicate. Finasteride concentrations were chosen based on a previous study, in which 10 µM and 50 µM induced significant metabolic modulation upon prostate tumoral cells [17]. Upon reaching 60% confluence, the cells were washed three times with sterile D-PBS (GIBCO/Invitrogen™), and the recommended culture medium (FBS free) was added as follows: 1) control treatment - supplemented with 0.1% DMSO; 2) low-dose finasteride treatment - supplemented with 0.1% DMSO plus 10 µM finasteride (Sigma™); and 3) high-dose finasteride treatment - supplemented with 0.1% DMSO plus 50 µM finasteride (Sigma™). Finasteride was diluted in DMSO, and the DMSO/finasteride solution was diluted in culture medium. During all finasteride exposure treatments, the cells were observed under a phase-contrast inverted microscope (Zeiss™) for general morphology and bacterial and fungal contamination. After 24, 48 and 72 hours of exposure, the conditioned medium (CM) was collected and individually stored at −80°C. The remaining attached cells were individually retrieved by trypsin digestion, and an aliquot was assessed for viability by Trypan Blue staining. The remaining cells were individually centrifuged at 1200 RPM for 10 minutes to obtain a cell pellet to produce protein extracts. FBS was removed during finasteride exposure because it is known to contain high levels of MMPs, which would interfere with the subsequent gelatinolytic assays.

### Conditioned Medium and Cell Extract Processing

The CM was concentrated 10X using Centriprep® 10,000 MWCO (Millipore™) tubes by centrifugation at 4900 RPM for 30 minutes. The respective cell pellets were submitted to protein extraction using an in-house-produced 50 mM Tris-HCl, 0.2 M NaCl, 10 mM CaCl_2_, 0.1% Triton X-100 protein extraction buffer supplemented with 1% protease inhibitor cocktail (Sigma-Aldrich™) to obtain cell extracts. The protein extraction buffer was chosen after comparison with a commercially available buffer (RIPA Buffer, Thermo Scientific™), which revealed better-preserved MMP gelatinolytic activity by zymography (data not shown). Protein extraction was conducted by repetitive pipetting and vortexing, followed by 1 hour of rest on ice and a final centrifugation at 5000 RPM for 20 minutes at 4°C. Finally, the CM and protein extracts were submitted to protein quantification using a NanoDrop 2000 (Thermo Scientific™) spectrophotometer and the Protein A280/260 protocol. It is important to emphasize that the CM was FBS free; therefore, all proteins in the CM were produced by the cells.

### MMP2 and MMP9 Activity Assays

The total (pro-form+active form) MMP2 and MMP9 activities in the CM and cell extracts were measured using the Biotrak® Activity Assays (GE Healthcare LifeSciences™) according to the manufacturer’s guidelines. Briefly, equal amounts (100 µg) of pooled CM or cell extract (per triplicate treatment) were pipetted into a 96-well microplate coated with anti-human-MMP2 or anti-human-MMP9 antibodies. Standards of human recombinant pro-MM2 or pro-MMP9 were pipetted into the respective wells, with values ranging from 0.19 to 3 ng/ml (for MMP2) and 0.125 to 4 ng/ml (for MMP9) as instructed by the manufacturer. After an overnight incubation at 4°C and washing, 50 µL of a 0.5 mM p-aminophenylmercuric acetate (APMA) solution was pipetted into all wells to activate the pro-forms of MMP2 and MMP9. Subsequently, 50 µL of a detection reagent (modified urokinase+S-2444™ peptide substrate) was added to all wells. The plates were incubated at 37°C for 6 (for MMP2) or 2 hours (for MMP9), and the integrated optical density (IOD) was measured at 405 nm using a spectrophotometric plate reader. Finally, a fold-change graphic was made by dividing the obtained IODs from the treated CM/extracts by their respective control values. Alternatively, the MMP2 and MMP9 activities were assessed using standard gelatin zymography, as previously described [16].

### TIMP-1 and TIMP-2 Protein Quantification

The TIMP-1 and TIMP-2 protein levels in the CM were measured using solid-phase ELISA Biotrak® Human Assays (GE Healthcare LifeSciences™) according to the manufacturer’s guidelines. Briefly, equal amounts (100 µg) of pooled CM (per triplicate treatment) were pipetted into a 96-well microplate coated with anti-human-TIMP-1 or anti-human-TIMP-2 antibodies. Standards of human recombinant TIMP-1 or TIMP-2 were pipetted into the respective plate wells, with values ranging from 3.13 to 50 ng/ml (for TIMP-1) and 8 to 128 ng/ml (for TIMP-2). After a 2 hour incubation at 25°C and washing, 100 µL of a peroxidase conjugate solution was pipetted into all wells, and the plates were further incubated for 2 hours at 25°C. Subsequently, 100 µL of a tetramethylbenzidine (TMB)/hydrogen peroxide solution was added to all wells. The plates were incubated at room temperature for 30 minutes, and the IOD was measured at a 630 nm using a spectrophotometric plate reader. Finally, a fold-change graphic was made by dividing the obtained IODs from the treated treatments by their respective control values.

### Migration Assay

The migration potential of all cell lines was investigated using the BD BioCoat™ Migration Insert (BD Biosciences™). The porous membrane (8 µm pore size) was hydrated with 500 µl of serum-free bicarbonate-based medium for 2 hours. After hydration, 500 µl of RPMI 1640 medium with 5% FBS (for LNCaP, PC3 and DU145 cells) or 500 µl of keratinocyte medium with 5% FBS (for RWPE-1 cells) was added to the lower chamber of the 24-well plate. These prostate cell lines were previously cultivated in 50 µM finasteride medium or control medium for 72 hours, harvested and individually cultivated in 200 µl serum-free medium (with or without 50 µM finasteride) in the insert at a density of 1×10^5^ total cells. The plates were incubated with 5% CO_2_ at 37°C for 22 hours according to the manufacturer’s guidelines. The cells that did not migrate, which were located on the top of the insert membrane, were scraped off with a cotton swab. The cells that migrated through the membrane pores, which were attracted by the FBS, were fixed in 100% methanol and stained with 0.1% toluidine blue solution for 2 minutes. The migrated cells were digitally photographed under 200X magnification using a light microscope (Nikon™). The experiments were performed in triplicate, and 5 random fields were photographed and subjected to cell counting using the ImageJ® free software. The migration index was calculated as the mean ± SD of cells counted per-field, and control cells were compared to finasteride-treated cells. Human skin fibroblasts (WS1– ATCC) were employed as positive controls and were exposed to the same assay conditions as the prostate cells.

### Wound Healing Assay

Cell migration was further investigated using a different migration assay for the PC3 and DU145 cell lines, which were cultivated in 6-well plates at 4×10^4^ cells/cm^2^ until 100% confluence was achieved. The monolayers were wounded (scratched) in a straight line across the well with a 200 µl pipette tip using a sterile, 6-well culture plate lid as a ruler, to obtain a regular scratch. The wounded monolayers were then washed twice with D-PBS (GIBCO/Invitrogen™) to remove cell debris. Culture medium with or without 50 µM of finasteride was added to the control and treatment wells. The wound area was subsequently inspected after 24, 48, 72 and 96 hours using an inverted phase contrast microscope with digital camera. The wound healing speed was calculated as the percentage of the initial wound until total wound closure at the different time-points using the ImageJ® free software.

### Matrigel Invasion Assay

The invasive potentials of all four cell lines were investigated using BD BioCoat™ Matrigel™ Invasion Inserts (BD Biosciences™). The matrigel-coated porous membrane (8 µm pore size) was hydrated with 500 µl of serum-free bicarbonate-based medium for 2 hours. After hydration, 500 µl of RPMI 1640 medium with 5% FBS (for LNCaP, PC3 and DU145 cells) or 500 µl of keratinocyte medium with 5% FBS (for RWPE-1 cells) was added to the lower chamber of the 24-well plate. These prostate cell lines were previously cultivated in 50 µM finasteride medium or control medium for 72 hours, harvested and individually cultivated in 200 µl of serum-free medium (with or without 50 µM finasteride) in the insert at a density of 1×10^5^ total cells. The plates were incubated in an incubator with 5% CO_2_ at 37°C for 22 hours, according to the manufacturer’s guidelines. The uninvaded cells on the top of the insert membrane were scraped off with a cotton swab. The cells that invaded the Matrigel matrix and migrated to the opposite side of the membrane, which were attracted by the FBS, were fixed in 100% methanol and stained with 0.1% toluidine blue solution for 2 minutes. The invaded cells were digitally photographed under 200X magnification using a light microscope (Nikon™). The experiments were performed in triplicate, and 5 random fields were photographed and subjected to cell counting using the ImageJ® free software. The invasion index was calculated as the mean ± SD of cells counted per-field, and control cells were compared to finasteride-treated cells.

### Western Blotting

The remaining cell extracts were used to determine AR expression levels. This determination was performed due to the discrepant literature on the expression of these receptors by these cell lines. Briefly, a protein sample (70 µg) that had been previously quantified using the NanoDrop 2000 (Thermo Scientific™) spectrophotometer was loaded onto a 10% SDS-PAGE gel under reducing conditions. The proteins were transferred onto a nitrocellulose membrane (Sigma Co™), which was subsequently blocked with 5% bovine serum albumin (BSA) in 10 mM Tris–HCl (pH 7.5), 150 mM NaCl and 0.1% Tween-20 (TBS-T) for 1 hour. The membranes were then incubated at 4°C overnight with the AR (Genetex™) antibody. The membranes were washed 5 times for 5 minutes in TBS-T and incubated for 2 hours at room temperature with the proper, peroxidase-conjugated secondary antibodies (ABCAM™). After washing in TBS-T, the membranes were visualized using 3,3′-diaminobenzidine and photographed.

### Statistical Analysis

The obtained data were analyzed using the INSTAT™ software using the two-tailed Student’s T test (P<0.05) to compare the different treatments and their respective controls (viability, migration, invasion), or using ANOVA and Dunnet’s post hoc test (MMPs and TIMPs assays).

## Results

### Finasteride Exposure and Cell Viability

Cell viability was not significantly affected by either of the finasteride doses that were employed in the present investigation. Cells did not present aspects of cell death or loss of viability, even after 72 hours of 50 µM finasteride exposure (Figure S1).

### MMP2 and MMP9 Activity Assays

**Figure 1:**
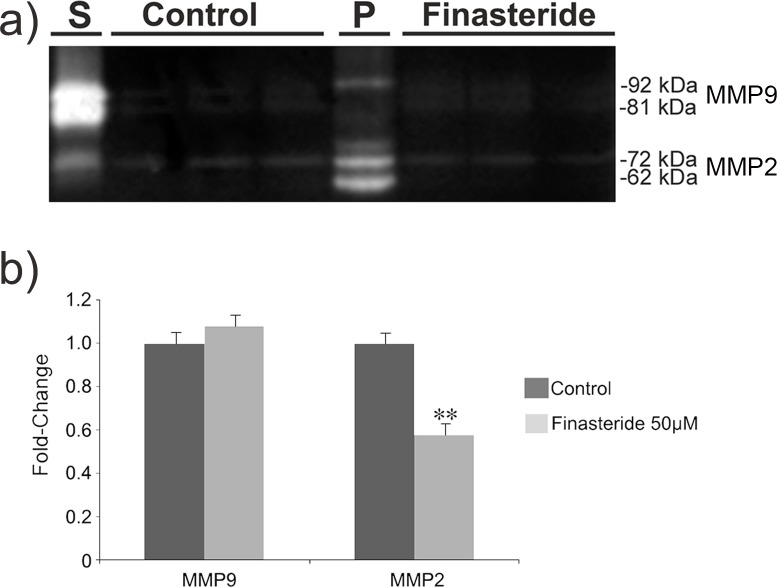
Finasteride’s effects on MMP2 and MMP9 activities in the conditioned medium of DU145 cells. **a)** Conditioned medium (CM) of untreated (Control) and finasteride-treated DU145 cells was collected, concentrated and analyzed for MMP2 and MMP9 activities using a gelatin zymography assay. (S) Standards, (P) Tissue extract of a gastric ulcer that was used as a positive control. These cells secreted high quantities of MMP2 and MMP9 into their CM, as shown by the bright bands on the dark background. **b)** High-dose finasteride (50 µM) downregulated MMP2 activity up to 43% after 72 hours of exposure, as quantified using the ImageJ™ software. Data are expressed as a fold-change graphic of the IOD values that were obtained from the bands in the (a) figure for finasteride-treated cells over control cells. (**) Statistically significant values with p<0.01. Experiments were carried out in triplicate.

In control conditions, it was observed that PC3 cells secreted twice as much MMP2 and MMP9 into their CM as RWPE-1 cells (IOD values – data not shown). LNCaP cells did not secrete detectable levels of MMP2 or MMP9 into their CM (IOD values data – not shown). However, DU145 cells secreted high quantities of MMP2 and MMP9 (Figure 1a). For the cell extracts, no cell line presented detectable MMP2 or MMP9 activities, demonstrating that these cell lines do not hold MMPs at their membrane (data not shown).

**Figure 2:**
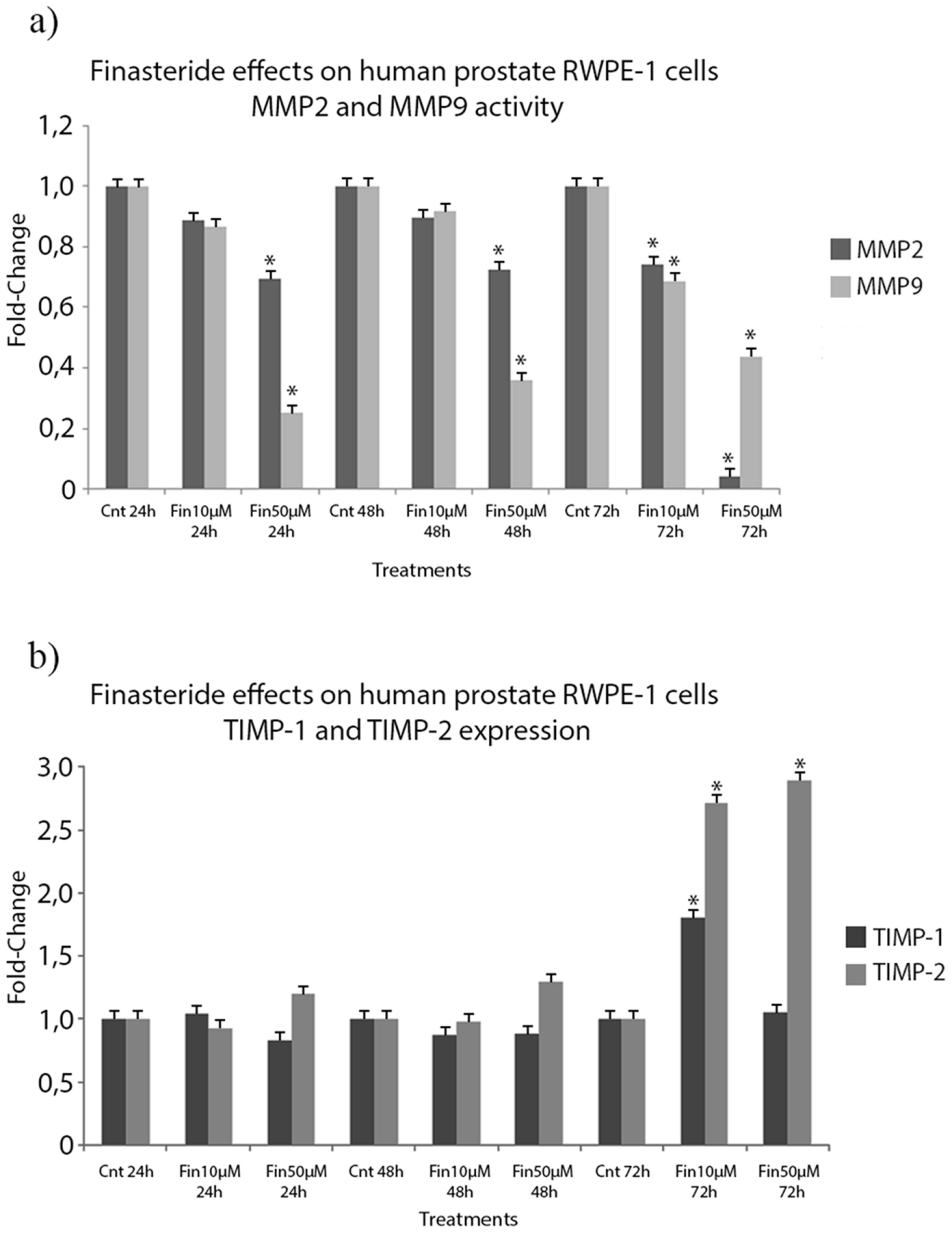
Finasteride’s effects on MMP2 and MMP9 activity and TIMP-1 and TIMP-2 expression in the conditioned medium of RWPE-1 cells. **a)** Conditioned medium of untreated (Control) and finasteride-treated RWPE-1 cells were collected, concentrated and analyzed for MMP2 and MMP9 activities using their respective Biotrak® Activity Assays. Low-dose finasteride (10 µM) for 72 hours of exposure downregulated MMP2 and MMP9 activity by 25% and 30%, respectively, when compared to control values. High-dose finasteride (50 µM) downregulated MMP2 and MMP9 activities at all tested time points, up to 90% and 55% after 72 hours of exposure, respectively. **b)** Conditioned medium of untreated (Control) and finasteride-treated RWPE-1 cells were collected, concentrated and analyzed for TIMP-1 and TIMP-2 protein expression using their respective Biotrak® Assays. Finasteride exposure, at both doses, induced the upregulation of TIMP-2 expression at the 72 hour time point, up to 150% more expression than control levels. Data are expressed as a fold-change graphic of the IOD values that were obtained for finasteride-treated cells over those of the control cells. (*) Statistically significant values with p<0.05.

Upon finasteride exposure, it was observed that, for RWPE-1 cells at the employed low dose (10 µM), 72 hours of exposure was sufficient to induce significant downregulation of both the activity of both MMPs in the CM (Figure 2a); these values were reduced by 25% and 30% for MMP2 and MMP9, respectively, relative to the control values. On the other hand, for the employed finasteride high dose (50 µM), the MMP2 and MMP9 activities were significantly downregulated at all time points that were tested, up to 90% and 55% after 72 hours of exposure, respectively (Figure 2a).

**Figure 3:**
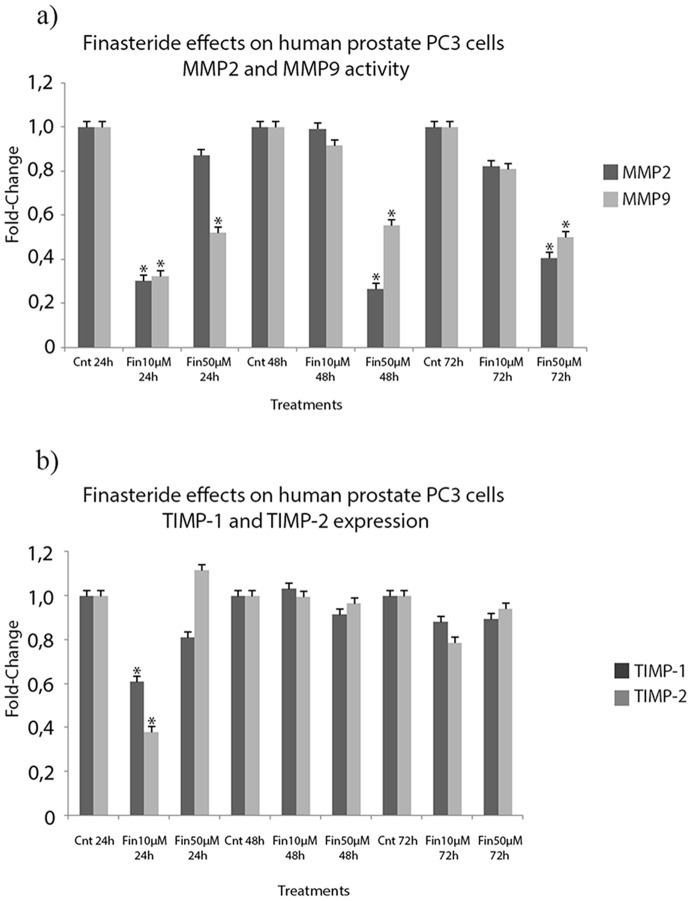
Finasteride’s effects on MMP2 and MMP9 activity and TIMP-1 and TIMP-2 expression in the conditioned medium of PC3 cells. **a)** Conditioned medium of untreated and finasteride-treated PC3 cells was collected, concentrated and analyzed for MMP2 and MMP9 activities using their respective Biotrak® Activity Assays. Low-dose finasteride exposure (10 µM) did not induce the downregulation of MMP2 or MMP9 activities, except at the 24 hour time point. High-dose finasteride induced the downregulation of MMP2 at the 48 hour and 72 hour time points and of MMP9 at all assessed time points, up to 70% reduction. **b)** Conditioned medium of untreated (Control) and finasteride-treated PC3 cells was collected, concentrated and analyzed for TIMP-1 and TIMP-2 protein expression using their respective Biotrak® Assays. Finasteride exposure did not induce any significant modulation of TIMP-1 and TIMP-2 expression, except for the 10 µM finasteride dose at 24 hours of exposure. Data are expressed as a fold-change of the IOD values obtained for finasteride treated cells over control cells. (*) Statistically significant values with p<0.05.

In the PC3 cell line, low-dose finasteride exposure induced the significant downregulation of MMP2 and MMP9 activities at only the 24 hour time point (Figure 3a). On the other hand, high-dose finasteride significantly induced the downregulation of MMP2 at the 48 hour and 72 hour time points and of MMP9 at all time points that were assessed, with up to a 70% reduction (Figure 3a).

Given that the results from the other cell lines showed that only the high-dose finasteride exerted effects on MMP activity, only the high dose at 72 hour was investigated for the DU145 cell line. For the DU145 cell line, high-dose finasteride induced a significant downregulation of MMP2 activities of approximately 43% (Figure 1b) (p<0.01). However, the MMP9 activities were not significantly modulated by finasteride exposure (Figure 1b).

### TIMP-1 and TIMP-2 Protein Quantification

When the cell lines were cultivated without finasteride, PC3 cells produced two times more TIMP-1 than the RWPE-1 cells; however, the amount of TIMP-2 was the same (IOD values). LNCaP cells produced low levels of TIMP-1 and TIMP-2 that neared the lower detection limit of the assay (IOD values). DU145 cells produced high quantities of TIMP-1 (IOD values – data not shown).

Upon finasteride exposure of the tested RWPE-1 cell line, both doses significantly induced upregulation of TIMP-2 expression at the 72 hour time point, which was up to 150% more expression than the control treatment (Figure 2b). As for PC3 cells, TIMP-1 and TIMP-2 expression showed no significant modulation, except for the 10 µM finasteride dose at 24 hours of exposure (Figure 3b). Finasteride did not induce any significant changes in the expression of TIMP-1 or TIMP-2 in LNCaP or DU145 cell lines (data not shown).

### Migration Assay

**Figure 4:**
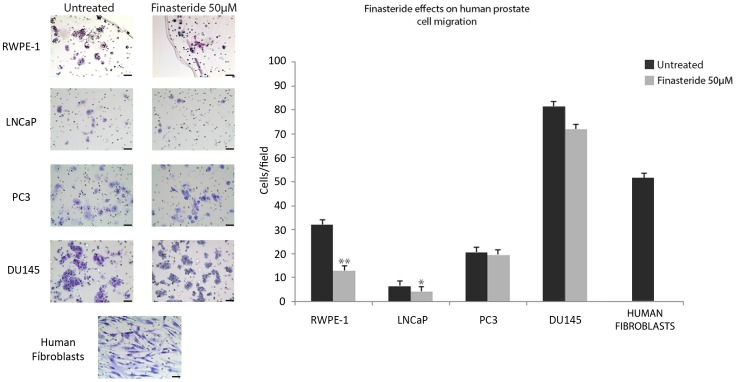
Finasteride’s effects on the migration of the human prostate cell lines RWPE-1, LNCaP, PC3 and DU145. The prostate cell lines were previously cultivated in 50 µM finasteride or control medium for 72 hours and individually cultivated in 200 µl of serum-free medium (with or without 50 µM finasteride) in the migration insert at a density of 1×10^5^ total cells. After 22 hours, cells that migrated through the 8 µm porous membrane were fixed, stained and counted within five random fields using a light microscope. Experiments were performed in triplicate. Representative photomicrographs of untreated and finasteride-treated cells are shown on the left side. Human fibroblasts (WS1 cell line) were employed as migration-positive control cells, and a representative image is shown on the bottom-left side. Scale bar = 40 µm. Finasteride significantly inhibited the cell migration of the RWPE-1 and LNCaP cell lines but not of the PC3 and DU145 cell lines. Data are expressed as the mean ± SD of the migrating cells. (*) Statistically significant values with p<0.05. (**) Statistically significant values with p<0.01.

In regular culture medium, the tested cell lines exhibited different migration potentials. RWPE-1 cells migrated more (mean of 32 cells/field) than PC3 cells (mean of 24 cells/field), which, in turn, migrated more than LNCaP cells (mean of 8 cells/field). DU145 was the most migrating cell line investigated (mean of 85 cells/field) (Figure 4). After 72 hours of finasteride exposure (50 µM), migration was inhibited significantly in RWPE-1 cells (p<0.01) and LNCaP cells (p<0.05). PC3 and DU145 cell migration was slightly inhibited; however, the inhibition was not significant (p>0.05) (Figure 4). Human WS1 fibroblasts (positive control) migrated promptly through the membranes (Figure 4).

### Wound Healing Assay

**Figure 5:**
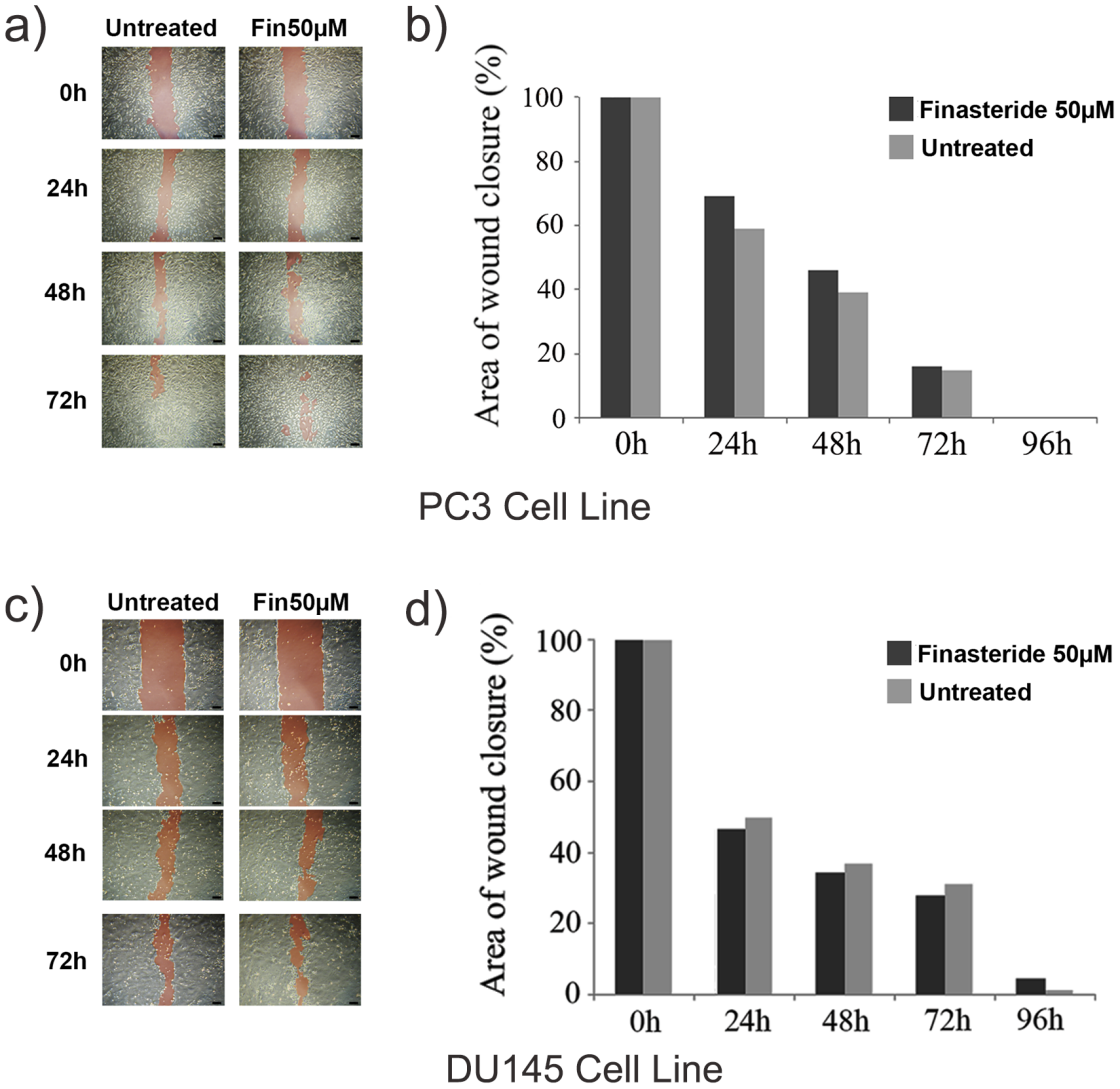
Finasteride’s effects on the migration of the human prostate cell lines PC3 and DU145 in the wound closure migration assay. Cells were cultivated in 6-well plates at 4×10^4^ cells/cm^2^ until 100% confluence was achieved. The monolayers were scratched in a straight line, and the wounded monolayers were then given culture medium with or without 50 µM finasteride. The wound area was inspected after 24, 48, 72 and 96 hours using an inverted phase contrast microscope with a digital camera. **a)** Representative images showing the wound closure of finasteride-treated and control PC3 cells at 0 to 72 hours. Red highlighted areas represent the open-wound area. Scale bar = 50 µm. **b)** The initial red highlighted areas were measured using the ImageJ™ software, and the remaining areas were calculated as a percentage of the initial wound area. A wound-closure graphic was made by dividing the area values that were obtained at the indicated time points for the control and finasteride-treated cells. Finasteride slightly inhibited cell migration; however, this difference was not statistically significant (p>0.05). **c)** Representative images showing the wound closure of finasteride-treated and control DU145 cells at 0 to 72 hours. Red highlighted areas represent the open wound area. Scale bar = 50 µm. **d)** As observed for the PC3 cells, finasteride slightly inhibited cell migration (p>0.05).

To confirm that the PC3 and DU145 cell line migration potentials were not influenced by finasteride exposure, a different migration assay was performed for these cell lines. After the wound area was inflicted upon the monolayers, it was possible to observe that, for both cell lines, finasteride slightly inhibited cell migration (Figure 5a and 5c); however, this difference was not significant (Figure 5b and 5d), which confirmed the results that were obtained from the previous migration insert assay (Figure 4).

### Matrigel Invasion Assay

**Figure 6:**
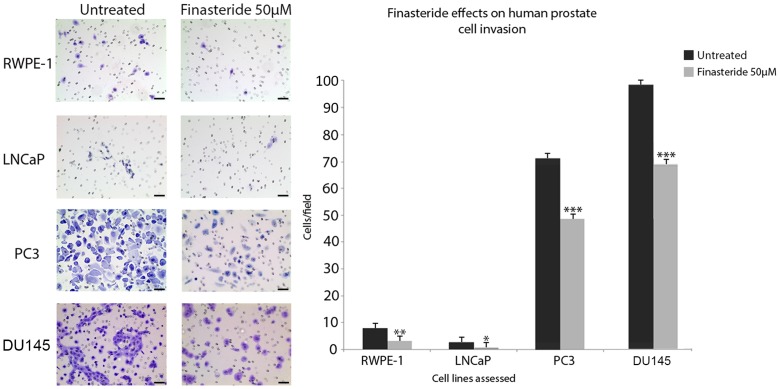
Finasteride’s effects on the invasion of the human prostate cell lines RWPE-1, LNCaP, PC3 and DU145. The prostate cell lines were previously cultivated in 50 µM finasteride or control medium for 72 hours and individually cultivated in 200 µl of serum-free medium (with or without 50 µM finasteride) in the Matrigel® invasion insert at a density of 1×10^5^ total cells. After 22 hours, cells that invaded through the matrigel matrix and porous membrane were fixed, stained and counted within five random fields using a light microscope. Experiments were performed in triplicate. Representative images of untreated and finasteride-treated cells are shown on the left side. Scale bar = 40 µm. Finasteride significantly inhibited the cell invasion of all tested cell lines. Data are expressed as the mean ± SD of the invading cells. (*) Statistically significant values, with p<0.05. (**) Statistically significant values, with p<0.01. (***) Statistically significant values, with p<0.001.

In regular culture medium, the cell lines exhibited different invasion potentials. DU145 and PC3 cells were the most invasive cell lines, with a means of 97 cells/field and 75 cells/field, respectively (Figure 6). The LNCaP cell line, which was also tumoral, curiously exhibited a low invasive potential, with a mean of 5 cells/field (Figure 6). The non-tumoral cell line RWPE-1 also exhibited low invasive potential, as expected, with a mean of 9 cells/field (Figure 6).

Finasteride inhibited cell invasion in all of the tested cell lines. DU145 and PC3 cell invasion was significantly reduced (p<0.001) followed by RWPE-1 (p<0.01) and LNCaP (p<0.05) (Figure 6).

### Western Blotting

**Figure 7:**
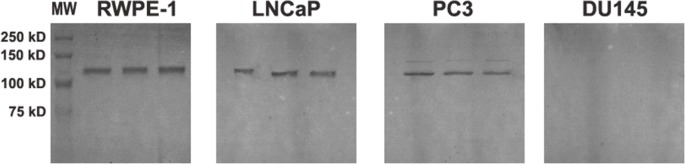
Western blots of whole protein extracts from RWPE-1, LNCaP, PC3 and DU145 cell lines. The prostate cell lines were cultivated in regular medium until 100% confluence and individually processed for protein extraction. After electrophoresis, the proteins were transferred onto a nitrocellulose membrane and probed with an anti-androgen receptor (AR) antibody. After incubation with the secondary antibody, the bands were visualized with DAB. All cell lines employed in this investigation expressed AR except for the DU145 cell line. Experiments were carried out in triplicate. (MW) Molecular Weight Standard. The estimated MW of AR is approximately 110 kDa.

AR was detected in the extracts of the LNCaP and RWPE-1 cell lines, as expected. However, PC3 cells, which are considered to be AR^−^, presented high expression of this receptor under our experimental conditions. DU145 cells did not express AR (Figure 7).

## Discussion

In this study, we evaluated the effects of different doses of finasteride on the prostate cells lines RWPE-1, LNCaP, PC3 and DU145 for different periods of exposure. Initially, we demonstrated that finasteride did not induce substantial cell death in any of the tested lines under any of the experimental conditions or techniques that were employed. Although it was previously reported that finasteride induced apoptosis in LNCaP cells [17], [18], the MTT technique, which was employed in both papers, is merely a proliferation assay. Although this method is suitable for anti-proliferative drug screening [19], it can lead to misleading data when employed as a viability assay without the proper backup assays. This was highlighted in some reports, in which the authors [4] first claimed that finasteride reduced the viability of LNCaP cells (using the MTT assay) and immediately claimed it reduced RWPE-1 cell growth (using the same MTT assay), which is an entirely different parameter. Therefore, although it is a very basic technique, trypan blue staining is more suitable for viability assessment because non-viable cells allow the dye to enter their membranes [20]. Moreover, published reports indicate that finasteride does not alter the expression profile of important proteins that are commonly used as apoptotic markers, such as caspase-3, insulin growth factor-1, Bcl-2, p53 and p21, and that its exposure leads to the downregulation of other apoptotic factors, such as caspase-7 and IGFBP-3, in prostate cancer samples from patients undergoing radical prostatectomy [21]. More evidence suggesting that finasteride might not always induce cell death was provided by Sawaya *et al*., (2002), who showed that its exposure decreased the levels of caspase that were detected in alopecia-treated patients when compared to non-affected individuals [22].

In subsequent experiments, we demonstrated that these cell lines produced low levels of MMPs in conditioned medium, even when more sensitive methods were used, such as those employed as a comparison to regular gelatin zymography. We also demonstrated that this activity was restricted in the CM because none of the tested cell lines exhibited MMP2 or MMP9 activities in their cell extracts. Interestingly, the obtained IODs from the control CMs did not correspond to the tumoral grading of these cell lines: although the most aggressive cell line, PC3, had greater MMP activity, the non-tumoral cell line, RWPE-1, had greater MMP activities than the tumoral cell line LNCaP. Additionally, we demonstrated that the DU145 cell line secretes more MMP2 and MMP9 than the other two tumoral cell lines that were investigated. It is important to note the variability in literature data about the levels, activity and gene expression of these metallopeptidases in these cell lines. Some authors have reported detectable gene expression of MMP7, MMP16, TIMP-1 and TIMP-2 in LNCaP and PC3 cells but no gene expression of MMP2 [23]. Other authors have reported that PC3 cells highly express MMP14 and MMP16, while LNCaP cells express them at lower levels [24]. On the other hand, MMP15 gene expression was reported to be higher in LNCaP cells than in PC3 cells [24]. Regarding protein expression, previous authors reported detectable levels of MMP1, MMP14, MMP15 and MMP16 by western blotting analyses in LNCaP and PC3 cells. TIMP-1 and TIMP-2 were detected, but at low levels [24]. On the other hand, low activities of MMP1, MMP7 and MMP13 were detected using gelatin zymography assays in the conditioned medium of these cell lines [24]. Our group has previously reported that when these cell lines are cultivated in regular culture medium, all display low activities of MMP2 and MMP9 in their CM [16]. We again observed these findings in the present report.

However, when the cells were cultured with diluted finasteride, PC3 and RWPE-1 cells exhibited significantly lower MMP2 and MMP9 activities in their CM. Furthermore, our results demonstrated that only high-dose finasteride (50 µM) was able to downregulate the MMP2 and MMP9 activities in these cell lines, as previously reported [16]. The activity of MMP2 in DU145 cells was also significantly inhibited by finasteride, which is a novel finding.

Interestingly, lower dose of finasteride downregulated MMP activities at the 24 hour time point in the PC3 cell line. At the 48 and 72 hour time points, the activities were normal, which we believe may be an adaptive response of the cells to the low-dose finasteride environment. The consistent downregulation of MMP activities that were induced by the higher-dose finasteride, through all experiments supports this hypothesis, as previously formulated in the normal/adaptive state/toxic state of cells/biological systems in response to different doses of a given antiandrogen [25]. On the other hand, RWPE-1 cells exhibited downregulated MMP2 and MMP9 activities at the 72 hour time point for the lower finasteride dose, indicating that this cell line has a limited ability to adapt compared to the PC3 cell line.

Based on these observations, it was expected that the expression of TIMP-1 and TIMP-2 could be modulated by finasteride because the activities of MMPs were regulated in this manner. We then proceeded to quantify the amounts of TIMP-1 and TIMP-2 in the CM of these cells under control and finasteride-treated conditions. Again, the LNCaP cell line did not express significant TIMP-1 or TIMP-2 in their CM. The expression of TIMP-1 was higher in PC3 cells than in RWPE-1 cells and similar to that in DU145 cells; however, the expression of TIMP-2 was similar in the CMs of both cell lines and control-treated cells. Although finasteride (50 µM) induced the downregulation of MMP activity in PC3, DU145 and RWPE-1 cells, this modulation might occur via different mechanisms in the cells, such as the upregulation of TIMP-1 and TIMP-2 in RWPE-1 cells or the reduced expression of MMPs by PC3 and DU145 cells. While these findings are relevant, one question is still unanswered: could finasteride induce high-grade prostate cancer? Therefore, the next experiment aimed to verify if this drug could directly modulate cell migration and invasion, which are important features related to tumor metastasis and, consequently, high-grade prostate cancer cases [26], [27].

Finasteride, when employed at a high dose, inhibited cell migration in the RWPE-1 and LNCaP cell lines but not in the PC3 and DU145 cell lines, in both migration assays. Interestingly, we found that RWPE-1 cells, which are classically defined as non-tumoral, had higher migration potential that the tumoral LNCaP cell line (compare RWPE-1 control cells to LNCaP control cells), which showed that this cell line did not display all of the characteristics that most authors would consider normal for this prostate cell line, given that tumoral cells should display high migration and invasion capabilities. In fact, previous authors have reported surprising results with this cell line, such as undetectable levels of nuclear AR, cytosolic SRD5A1, and nuclear SRD5A2 proteins using western blotting techniques [4]. Although cell motility is an important feature for the success of cells to metastasize, cell invasion is mandatory for tumor cells to migrate through the ECM [10], [12], [16] to gain access to blood or lymph vessels and ultimately reach circulation. We exposed all cell lines to 50 µM finasteride for 72 hours and then subjected them to another 22 hours of finasteride exposure during the transwell-Matrigel™ invasion assay. Initially, we again observed surprising results for RWPE-1 cells, such as the ability of cell invasion. This finding and the previously described high migration potential of this cell line support the concern that has been raised regarding RWPE-1 cell line physiology and non-tumoral status [37]. We have also demonstrated and observed that untreated LNCaP cells have approximately one-tenth of the invasion potential of untreated PC3 cells, as reported previously [27], [28], and, more importantly, our results clearly demonstrate that finasteride significantly inhibits prostate cancer cell invasion in all four tested cell lines. Others studies using vascular smooth muscle cells (AR^+^) and fibroblasts (AR^+^) have reported the consistent inhibition of migration speed by flutamide and bicalutamide, respectively [29], [30]. Although the cells were exposed to finasteride in FBS-free medium in the present study, these cell lines were able to synthesize T even in an FBS-starved medium environment by acquiring complete steroidogenic ability to produce androgens [31]. Thus, our findings confirm those of previous reports [29]–[30] showing that DHT stimulates cells to migrate and invade more than T, which does not occur on a finasteride-rich microenvironment.

Intriguingly, it is well known and reported that these four cell lines present different integrities of the androgenic signaling machinery, mainly in terms of AR mRNA and protein expression [4], [32]–[37]. Finasteride treatment induced a similar result of reduced Matrigel™ invasion ability by these cell lines, and further complicating this issue, our PC3 cells presented strong bands for AR. Although being classically considered as AR^−^, some previous reports have demonstrated that PC3 and DU145 express this receptor [35]. Even more intriguing, other authors report that descendent PC3 cell lines, which were originally obtained from the ATCC supplier, and expanded more than 70 passages from receipt, had detectable levels of AR mRNA, being classified as PC3^AR+^ [36]. We hypothesize that recent reports, which mainly used over-passaged cell lines, may have failed to observe this change in AR mRNA expression given that the early classical studies had already reported this cell line as AR^−^.

Therefore, although it is accepted that AR may play a role in the regulation of cell migration and invasiveness [30], [38], [39], similar results showing the downregulation of these features after finasteride exposure in cell lines that express or do not express this receptor suggest that this effect may be due to a non-canonical mechanism of action of finasteride in these cells, which remains to be elucidated.

## Conclusion

Although the debate regarding the higher incidence of high-grade prostate cancer among 5-ARI-treated patients remains, our findings indicate that finasteride may attenuate tumor aggressiveness and invasion, which could vary depending on the phenotypes of the patients’ prostate cells, mainly the cell’s androgen responsiveness status. Although these results may seem beneficial for finasteride-treated patients, future studies are necessary to elucidate the mechanism by which finasteride exerts its effects on prostate tumoral cells.

## Supporting Information

Figure S1 **Representative images of human prostate cells general morphology and cell density (cells/area) under control conditions, and after 72 h of finasteride exposure (50 µM).** Prostate cell lines RWPE-1, LNCaP, PC3, and DU-145 were plated at 2×10^4^cels/cm^2^, cultivated until reaching 60% confluence, and exposed to finasteride. General morphology was similar between untreated and finasteride-treated cells. No significant apoptotic or necrotic morphological characterists were observed. Scale bar = 100 µm.
(TIF)
